# Differences in smokers’ awareness of the health risks of smoking before and after introducing pictorial tobacco health warnings: findings from the 2012–2017 international tobacco control (ITC) Netherlands surveys

**DOI:** 10.1186/s12889-020-08667-9

**Published:** 2020-05-08

**Authors:** Dirk-Jan A. van Mourik, Gera E. Nagelhout, Marc C. Willemsen, Bas van den Putte, Hein de Vries

**Affiliations:** 1 Department of Health Promotion, Maastricht University (CAPHRI), PO Box 616, 6200 MD Maastricht, the Netherlands; 2 Department of Family Medicine, Maastricht University (CAPHRI), Maastricht, the Netherlands; 3 IVO Research Institute, The Hague, the Netherlands; 4 Netherlands Expertise Center for Tobacco Control (NET), Trimbos Institute, Utrecht, the Netherlands; 5 Department of Communication, University of Amsterdam, Amsterdam, the Netherlands

**Keywords:** Awareness, Longitudinal, Smokers, Pictorial health warnings, The Netherlands

## Abstract

**Background:**

As of May 2016, pictorial health warnings (PHWs) showing the harms of smoking were implemented in the European Union. After one year they had to be fully implemented. We studied changes in awareness of the health risks of smoking after implementation of PHWs among smokers from the Netherlands, whether the trend before the implementation changed after the implementation, and whether there were differences between subgroups.

**Methods:**

We used survey data from six yearly waves of the International Tobacco Control (ITC) Netherlands Survey from 2012 to 2017. The number of participating smokers ranged between 1236 and 1604 per wave. Data were analyzed using Generalized Estimating Equations (GEE) analyses.

**Results:**

Indicators of awareness of the health risks of smoking that did not change between 2015 and 2017 were perceived susceptibility (β = 0.043, *p* = 0.059) and perceived severity (β = − 0.006, *p* = 0.679) regarding lung problems. Perceived susceptibility, however, was more pronounced between 2015 and 2017 than between 2012 and 2015(*p* value of interaction: *p* = 0.044). Noticing information about the dangers of smoking (β = 0.119, *p* < 0.001) and knowledge about the health risks of smoking (β = 0.184, p < 0.001) increased between 2015 and 2017. These increases were both more pronounced when compared to 2012–2015 (*p* values of interactions: *p* = 0.002 and p < 0.001 respectively). Compared to high educated smokers, low educated smokers (β = − 1.137, p < 0.001) and moderate educated smokers (β = − 0.894, p < 0.001) were less knowledgeable about the health risks of smoking in 2016 and 2017.

**Conclusions:**

Introducing PHWs coincided with an increase in smokers’ knowledge about the health risks of smoking. Dutch tobacco control policy and campaigns should focus on improving Dutch smokers’ awareness of the health risks of smoking even more, especially among low educated smokers.

## Background

Pictorial health warnings (PHWs) on the packet of tobacco products [1] were introduced as of May 2016 in the European Union (EU) as part of the second Tobacco Products Directive (TPD) (2014/40/EU) [2]. The PHWs are printed on 65% of the front and the back of the packet. Also, the packet features textual health warnings (THWs) next to the PHWs and on 50% of its lateral sides. The Netherlands solely used THWs before the introduction of the second TPD, which had to be fully implemented as of May 2017. Table 1 provides an overview of the THWs before [3, 4] and after the second TPD in the Netherlands [1, 2].

**Table 1:** Textual health warnings and efficacy messages from the first and second Tobacco Products Directive (TPD) on the packet of tobacco products

Textual health warning (THW) in first TPD	Comparable THW in second TPD
Smoking causes blockage of the blood vessels, heart attacks, and strokes	Smoking clogs your arteries Smoking causes heart attacks Smoking causes strokes and disability
Smoking causes deadly lung cancer	Smoking causes 9 out of 10 lung cancers Smoking damages your lungs Tobacco smoke contains more than 70 substances that cause cancer^b^
Quitting smoking reduces the risk of fatal heart and lung diseases	Smoking causes heart attacks Smoking causes 9 out of 10 lung cancers Smoking damages your lungs
Smoking during pregnancy is unhealthy for your baby	Smoking can kill your unborn child
Protect children: do not let them breathe your smoke Smoking causes serious harm to you and others around you^a^	Your smoke harms your children, family and friends
Smoking can reduce blood circulation and cause impotence	Smoking increases the risk of impotence
Smoking can damage the sperm and reduces fertility	Smoking reduces fertility
Smoking is deadly^a^	Smoking is deadly – quit now^b^
Smokers die younger	-
Tobacco smoke contains benzene, nitrosamines, formaldehyde and hydrogen cyanide	-
Smoking ages the skin	-
Your doctor or pharmacist can help you quit smoking	-
Smoking is very addictive; do not start	-
Smoking can lead to a slow, painful death	-
-	Smoking causes mouth and throat cancer
-	Smoking damages your teeth and gums
-	Smoking increases the risk of blindness
-	Smokers’ children are more likely to start smoking
-	Quit smoking – Stay alive for those close to you
**Efficacy message**	
Find help to stop smoking: DEFACTO 0900–9390 (€ 0.10 / min) or www.stoppen-met-roken.nl or consult your doctor or pharmacist	Quit now! Go to www.ikstopnu.nl. Or call the quit line 0800–1995 (free)

^a^One of two general warnings that were placed on each packet on 30% of the front of the packet. The other warnings were placed on 40% of the back of the packet

^b^One of two general warnings that were placed on each packet on 50% of the lateral sides

Tobacco health warnings communicate information about the health risks of smoking. This intervention is a method of ‘consciousness raising’ to influence awareness as the warnings communicate the consequences of smoking. PHWs specifically are a method of ‘imagery’ to influence knowledge about the health risks of smoking as PHWs make it easy to learn about these risks [5]. Smokers who know about the health risks are more likely to intend to quit smoking [6–9]. Also, many health behavior theories predict that knowledge about the health risks of the behavior precedes behavior change (e.g. quitting smoking) [10–13]. The current study aims to examine whether Dutch smokers’ knowledge about the health risks of smoking was different after the introduction of PHWs in 2016. Previous studies from Australia [14–18], England [19], Mexico [20], Taiwan [21], and Thailand [22] found increases in smoking related knowledge among smokers after the change from THWs to PHWs. However, the impact of the EU PHWs on knowledge about the health risks of smoking has not been assessed yet.

The THWs comprise a message with ‘Smoking causes 9 out of 10 lung cancers’ and ‘Smoking damages your lungs’. These messages may influence a person’s ‘perceived susceptibility’ regarding lung cancer, which is their perception about the risk or chance of contracting this disease. Such perceptions may play a role in smoking cessation as smokers who are feeling more susceptible to these health risks more often intend to quit smoking [23–25]. Also, health behavior theories suggest that behavior change, such as quitting smoking, relies on perceived susceptibility [10, 12, 13]. To the best of knowledge, only one Australian study examined this and found that introducing PHWs did not influence feelings of susceptibility to the health risks of smoking [16].

Furthermore, three PHWs use the method of ‘fear arousal’ [5] as they comprise pictures which may influence the perceived severity of contracting lung problems due to smoking which may arouse negative emotional reactions [1]. Perceived severity concerns beliefs about the significance or magnitude of the health risk of smoking. Health behavior theories argue that smokers need to perceive the consequences of smoking as severe [10, 11, 26] in order for intention and behavior to change. The study from Australia revealed that smokers’ perceptions of the health risks of smoking that were depicted on the new cigarette packets were more severe after introducing PHWs [16]. The current study aims to examine whether Dutch smokers’ risk perceptions (perceived susceptibility and perceived severity) were different after the introduction of PHWs in 2016.

According to the I-Change model, knowledge forms a person’s awareness of the health risks of smoking, together with risk perception, and noticing advertising or information about the dangers of smoking (perceived cues) [11]. To date, no studies examined if perceived cues among smokers changed after introducing PHWs. This study aims to fill this gap.

Before the introduction of PHWs, Dutch campaigns only focused on positioning non-smoking as the social norm, quitting smoking, and prevention of smoking. Therefore, the current study aims to examine the trend in harm awareness between 2012 and 2015 and whether this trend differs from the trend between 2015 and 2017. This study may provide insights in what happens with smoker’s awareness about the health risks of smoking when there are no policies that aim to improve awareness.

This paper further aims to identify subgroup differences in awareness of the health risks of smoking. In order to target campaigns at the most relevant subgroups of smokers, it is important to explore differences according to age, education, and gender. Previous research showed that lower education is associated with less knowledge about the health risks of smoking [6, 7, 9, 27–30]. Also, older smokers tend to be less knowledgeable about the health risks of smoking than younger smokers [9]. However, it is unknown whether and – if so – how Dutch smoker subgroups differ in their awareness of the health risks of smoking.

In sum, the current study was designed to examine three research questions: (1) Did smokers show changes in awareness of the health risks of smoking after introducing PHWs in 2016? (2) Did the trends in awareness of the health risks of smoking after introducing the EU’s PHWs differ from the trends before their introduction; and (3) Did awareness of the health risks of smoking in 2016 and 2017 differ by age, educational level, and gender? Results from our study may result in recommendations for future policy regarding health warnings and campaigns on the national and European level.

## Methods

### Sample

This study is part of a larger PhD Project on the evaluation of tobacco control policies in the Netherlands; previously published papers from this PhD Project have used the same dataset but explore different research questions [31, 32]. Data were derived from the International Tobacco Control (ITC) Netherlands Waves 6–11 Surveys. Information regarding data collection, ethics clearance, incentives for participation, and inclusion criteria can be found elsewhere [31, 32]. To compensate for attrition effects, sampling weights and tailored replenishment samples were used [33].

**Fig. 1:**
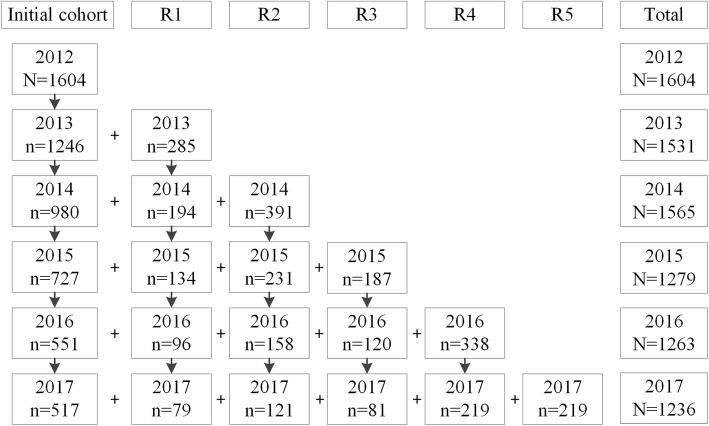
Recruitment-flowchart of smokers in the International Tobacco Control Netherlands Survey between 2012 and 2017*. *R, replenishment

For our analyses, data from survey Wave 6 (May to June 2012; *N* = 2022), Wave 7 (May to June 2013; *N* = 1970), Wave 8 (May to June 2014; *N* = 2008), Wave 9 (November to December 2015; *N* = 1720), Wave 10 (November to December 2016; *N* = 1696; shortly after the implementation of the EU’s PHWs as of May 2016), and Wave 11 (November to December 2017; N = 1696; the PHWs were fully implemented as of May 2017) were used. Those who quit smoking were excluded from all analyses because they did not receive all questions and awareness of the health risks of smoking tends to change after people quit smoking. Between survey waves, between 12.6 and 29.3% of the respondents per wave were excluded from the analyses because they had quit smoking. Attrition among smokers ranged from 17.8 to 25.4% between survey waves. Figure 1 shows each survey wave’s number of smokers.

### Measures

Information regarding the measures on perceived cues, risk perception (perceived susceptibility and perceived severity), knowledge about the health risks of smoking, moderators (education, gender, and age), and control variables (Heaviness of Smoking Index, quit intention, and ever-quit) can be found elsewhere [31]. Concerning knowledge about the health risks of smoking, the eight items combined showed a good reliability with a Cronbach’s Alpha ranging between α = 0.832 and α = 0.878 over the years.

### Statistical analysis

Data were analyzed using SPSS 23.0. To ensure representativeness of the sample, this study’s tests and statistical estimates were weighted by age and gender [34]. For the first research question, whether Dutch smokers showed changes in awareness of the health risks of smoking after introducing PHWs in 2016, Generalized Estimating Equations (GEE) analyses over the years 2015 to 2017 were employed. For the second research question, whether the trend in awareness about the health risks of smoking differed before and after introducing the EU’s PHWs, we used data from 2012 to 2017 and added interaction terms between the intervention factor (the introduction of PHWs) and Wave to the GEE analyses. We created an intervention factor by coding the years 2012–2015 as ‘0’ and the years 2016–2017 as ‘1’. In addition, we employed GEE analyses over the years 2012 to 2015. To answer the third research question, differences between subgroups were determined in a GEE model including the two most recent survey waves (2016–2017). All control and moderating variables were included in all models. Moreover, the analyses were adjusted for time-in-sample (the number of times a respondent participated in the cohort) as this may influence responses [35]. The repeated measure variable was survey wave. Only for the individual knowledge items the binominal distribution and the logit link were used, while for all other measures the normal distribution and the identity link were used. The unstructured correlation structure was used for all GEE analyses [36]. To correct for multiple testing, only results with a *p*-value of < 0.01 were considered as statistically significant [37].

## Results

### Sample characteristics

Table 2 shows sample characteristics of smokers between 2012 and 2017. The sample differed significantly over the years on all sample characteristics.

**Table 2:** Characteristics of smokers between 2012 and 2017^*^

	2012 (*n* = 1604)	2013 (*n* = 1531)	2014 (*n* = 1565)	2015 (*n* = 1279)	2016 (*n* = 1263)	2017 (*n* = 1236)	
**Age group**							
15–24 years (%)	13.7	11.5	11.1	15.5	11.5	10.6	χ^2^ = 44.931
25–39 years (%)	23.5	23.7	23.8	24.1	21.1	22.0	p < 0.001
40–54 years (%)	32.1	32.2	29.8	27.9	30.0	29.8	
55 years and older (%)	30.7	32.6	35.3	32.6	37.4	37.5	
**Educational level**							
Low (%)	31.8	29.1	26.1	24.7	23.7	23.7	χ^2^ = 71.467
Moderate (%)	44.9	46.1	43.7	42.4	44.8	44.8	p < 0.001
High (%)	23.3	24.8	30.2	33.0	31.5	31.5	
**Gender**							
Male (%)	54.1	52.0	50.7	54.0	59.4	59.5	χ^2^ = 37.729
Female (%)	45.9	48.0	49.3	46.0	40.6	40.5	p < 0.001
**Ever tried to quit**							
Yes (%)	60.9	60.2	57.8	64.2	70.7	73.5	χ^2^ = 114.915
No (%)	39.1	39.8	42.2	35.8	29.3	26.5	p < 0.001
**Heaviness of Smoking Index**							
0–1 (%)	28.0	28.7	29.4	31.7	36.7	37.0	χ^2^ = 59.74
2–4 (%)	64.5	64.4	64.5	62.2	58.5	58.9	p < 0.001
5–6 (%)	7.5	6.9	6.0	6.2	4.8	4.1	
**Quit intentions** (mean^a^, SD))	2.6 (1.1)	2.6 (1.2)	2.5 (1.2)	2.6 (1.2)	2.7 (1.2)	2.75 (1.29)	F = 8.778
							p < 0.001
**Smoking frequency**							
Daily (%)	92.5	91.2	90.6	91.5	90.7	91.2	χ^2^=29.354
Weekly (%)	4.8	6.5	6.3	4.3	5.4	4.3	p=0.001
Monthly (%)	2.7	2.3	3.0	4.2	3.9	4.5	

*SD* standard deviation

*Estimates were weighted for gender and age. χ^2^ refers to the result of chi-square analyses while the F-value refers to the results of independent sample t-tests

^a^: On a scale from 1 to 5

### Perceived cues

Table 3 shows that the mean score on perceived cues increased from 2.3 to 2.7 over the years. The increase between 2012 and 2017 was significant as shown by the GEE analysis from Table 4. A significant interaction term between the intervention factor and Wave was found (*p* = 0.002). Separate GEE analyses showed that between 2012 and 2015 there was no change in perceived cues, while a significant increase was found between 2015 and 2017 (Table 4).

**Table 3:** Level of perceived cues, perceived susceptibility, perceived severity, and knowledge about the health risks of smoking by year between 2012 and 2017^a^

	2012	2013	2014	2015	2016	2017
Perceived cues (mean^b^, (SD))	2.3 (0.9)	2.4 (1.0)	2.3 (1.0)	2.4 (1.0)	2.7 (1.0)**	2.7 (1.1)
Perceived susceptibility (mean^b^, (SD))	3.1 (0.9)	3.1 (1.0)	3.1 (0.9)	3.2 (1.0)	3.2 (1.0)	3.2 (1.0)
Perceived severity (mean^b^**,** (SD))	3.4 (1.0)	3.4 (1.0)	3.3 (1.0)	3.3 (1.0)	3.6 (1.1)**	3.5 (1.0)
Knowledge (mean^c^, (SD))	4.3 (2.2)	4.3 (2.3)	4.4 (2.3)	4.5 (2.4)	4.8 (2.5)**	5.1 (2.6)**
Blindness (%)	7.7	9.5	11.7	12.0	23.4**	34.6**
Heart disease (%)	74.3	73.9	74.6	73.1	77.6*	78.9
Impotence (%)	50.5	52.3	54.7	54.0	56.5	59.1
Lung cancer (%)	86.1	86.2	86.1	84.3	86.0	85.5
Mouth and throat cancer (%)	75.1	73.5	72.9	74.1	77.1	80.6
Stroke (%)	62.0	60.6	60.1	59.6	65.0*	67.4
Lung cancer due to SHS (%)	53.7	52.0	52.4	56.0	53.0	57.5
Heart disease due to SHS (%)	38.1	38.2	38.6	41.0	44.1	48.2

SD, standard deviation

SHS, secondhand smoke

^a^: Estimates were weighted for gender and age

^b^: On a scale from 1 to 5

^c^: On a scale from 0 to 8

*Paired sample t-test or χ^2^ analysis showed a difference of *p* < 0.01 compared with the previous wave

**Paired sample t-test or χ^2^ analysis showed a difference of *p* < 0.001 compared with the previous wave

**Table 4:** Regression coefficients and odds ratios of Generalised Estimation Equations of perceived cues, perceived susceptibility, perceived severity, and knowledge^a,b^

	2012–2017 β (95% CI)	2012–2015 β (95% CI)	2015–2017 β (95% CI)	*p*-value^**c**^
Perceived cues	0.052 (0.013 to 0.091)*	−0.026 (−0.067 to 0.015)	0.119 (0.066 to 0.172)**	*p* = 0.002
Perceived susceptibility	0.014 (−0.032 to 0.059)	0.010 (−0.033 to 0.053)	0.043 (−0.002 to 0.088)	*p* = 0.044
Perceived severity	0.015 (−0.025 to 0.054)	− 0.010 (− 0.052 to 0.032)	−0.006 (− 0.032 to 0.021)	*p* = 0.764
Knowledge – sum score	0.194 (0.090 to 0.299)**	0.116 (0.028 to 0.203)*	0.184 (0.088 to 0.280)**	*p* < 0.001
Blindness	1.465 (1.309 to 1.641)**	1.250 (1.097 to 1.425)*	1.867 (1.645 to 2.120)**	*p* < 0.001
Heart disease	1.116 (1.013 to 1.229)	1.113 (1.013 to 1.222)	1.114 (0.994 to 1.249)	*p* = 0.051
Impotence	1.185 (1.085 to 1.294)**	1.106 (1.018 to 1.200)	1.183 (1.076 to 1.302)*	*p* < 0.001
Lung cancer	0.907 (0.803 to 1.025)	1.087 (0.965 to 1.226)	0.910 (0.802 to 1.033)	*p* = 0.053
Mouth and throat cancer	1.121 (1.103 to 1.241)**	1.136 (1.033 to 1.249)*	1.178 (1.047 to 1.327)*	*p* = 0.031
Stroke	1.170 (1.074 to 1.275)**	1.107 (1.017 to 1.205)	1.112 (1.007 to 1.226)	*p* = 0.001
Lung cancer due to SHS	1.022 (0.941 to 1.108)	0.976 (0.900 to 1.060)	1.050 (0.960 to 1.150)	*p* = 0.095
Heart disease due to SHS	1.080 (0.996 to 1.171)	1.042 (0.958 to 1.132)	1.159 (1.055 to 1.273)*	*p* = 0.106

*SHS* secondhand smoke

^a^: Estimates were weighted for age, and gender

^b^: Analyses were controlled for age group, gender, educational level, level of addiction to tobacco, intention to quit smoking, and ever having made a quit attempt

^c^: Of the interaction term between the intervention factor (introduction of PHWs) and Wave, in the GEE analysis over the years 2012–2017

*p < 0.01, **p < 0.001

Smokers who were more likely to perceive cues to quit smoking were 55+ smokers compared to aged 15–24 smokers (β = − 0.344; Confidence Interval (CI) = − 0.547 to − 0.122; *p* = 0.002), aged 40–54 smokers compared to aged 15–24 smokers (β = − 0.297; CI = − 0.516 to − 0.077; *p* = 0.008), and female smokers compared to male smokers (β = 0.177; CI = 0.056 to 0.299; *p* = 0.004).

### Risk perception

The mean score for perceived susceptibility to developing lung cancer fluctuated between 3.1 and 3.2 over the years, while the mean score for perceived severity for developing lung problems fluctuated between 3.4 and 3.6 (Table 3). As shown in Table 4, the changes in perceived susceptibility and in perceived severity were not significant. The interaction term between the intervention factor and Wave was significant for perceived susceptibility (p = 0.002) while no significant interaction term was found for perceived severity (*p* = 0.764). This implies that for perceived susceptibility, the increase between 2016 and 2017 was significantly stronger when compared to the increase between 2012 and 2015.

The subgroup more likely to feel susceptible to developing lung cancer was aged 40–54 smokers compared to aged 55+ smokers (β = 0.278; CI = 0.139 to 0.417; *p* < 0.001), and aged 40–54 smokers compared to aged 25–39 smokers (β = − 0.244; CI = − 0.380 to − 0.068; *p* = 0.005). Subgroups more likely to perceive developing lung problems as severe were aged 25–39 smokers compared to aged 55+ smokers (β = 0.264; CI = 0.108 to 0.421; *p* = 0.001), and aged 40–54 smokers compared to aged 55+ smokers (β = 0.314; CI = 0.182 to 0.447; *p* < 0.001).

### Knowledge about the health risks of smoking

Dutch smokers’ scores on knowledge increased from 4.3 in 2012 to 5.1 in 2017 (Table 3). The GEE analysis from Table 4 reveals that overall knowledge about the health risks of smoking increased between 2012 and 2017. The increase was more pronounced between 2015 and 2017 than between 2012 and 2015: there was a significant interaction term between the intervention factor and Wave (*p* < 0.001). The increases in knowledge about smoking causing blindness and impotence in male smokers, mouth and throat cancer, and secondhand smoke causing heart disease were more pronounced between 2015 and 2017 than between 2012 and 2015. However, only the CI’s of the trends of knowledge about smoking causing blindness were not overlapping. No changes were found in knowledge about smoking causing heart disease, lung cancer, and secondhand smoke causing lung cancer.

Subgroups more likely to be knowledgeable about the health risks of smoking were aged 25–39 smokers compared to aged 55+ smokers (β = 0.764; CI = 0.383 to 1.145; *p* < 0.001), and aged 40–54 smokers compared to aged 55+ smokers (β = 0.534; CI = 0.241 to 0.827; p < 0.001). Also, high educated smokers were more likely to be knowledgeable than low educated smokers (β = − 1.137; CI = − 1.499 to − 0.776; p < 0.001) and moderate educated smokers (β = − 0.894; CI = − 1.207 to − 0.581; p < 0.001).

## Discussion

Concerning the first research question, to examine if Dutch smokers showed changes in awareness of the health risks of smoking after introducing PHWs in 2016, we found an increase in knowledge about the health risks of smoking. This finding indicates that PHWs could be an effective method of ‘consciousness raising’ and ‘imagery’ [5]. Our explanation for this finding is that the introduction of PHWs made smokers more often notice the health warnings [38, 39]. Another explanation may be that the EU’s PHWs communicated new facts that were not extensively communicated before, especially that smoking increases the risk of blindness [1, 2]. For perceived severity and susceptibility, no changes over time were found. Perhaps the message ‘Smoking causes 9 out of 10 lung cancers’ may not influence beliefs about the risk or chance of contracting lung cancer due to smoking as smokers may not be aware of the incidence of this disease. Furthermore, there was no increase in perceived severity. Possibly smokers already perceived lung problems due to smoking as serious and the PHWs did not make these beliefs more salient.

Concerning the second research question we found a stronger increase in knowledge about the health risks of smoking after the introduction of the PHWs than before. The data also showed that only after introducing PHWs there was an increase in Dutch smokers noticing advertising or information that talks about the dangers of smoking, or encourages quitting. Our explanation is that between 2012 and 2015 Dutch campaigns only focused on positioning non-smoking as the social norm, quitting smoking, and prevention of smoking. This implies that Dutch tobacco policy did not meet Article 12 of the World Health Organization (WHO) Framework Convention on Tobacco Control (FCTC), which requires ratifying countries to promote public awareness of and access to information regarding the health risks of smoking [40]. Another explanation is that the Netherlands solely used THWs on the packet of tobacco products until PHWs were introduced.

Concerning the third research question, to identify subgroup differences in awareness of the health risks of smoking, we found that, in line with previous research [9], younger smokers were more knowledgeable about the health risks of smoking than older smokers. Younger generations may be better educated about the health risks of smoking. Another explanation may come from the cognitive dissonance theory [41]. More for older than for younger smokers, their actions (smoking) may be in conflict with their knowledge (about the health risks of smoking). Their chances of being harmed due to smoking are higher because their older age makes them more likely to have smoked more cigarettes in their lifetime. This conflict may lead to beliefs that discount the health risks of smoking [42]. This finding might also explain why older smokers perceived developing lung problems as less serious compared to younger smokers. Also, high educated smokers were more knowledgeable about the health risks of smoking than low educated smokers, in line with previous research [6, 7, 9, 27–30], which may have been caused by more frequent exposure to health information, better understanding of health information, or they may be better trained to obtain health information.

### Limitations

Differences in sample characteristics were found over the years (Table 1). In response to this, the analyses were adjusted for the sample characteristics, we applied sampling weights, and adjusted for time-in-sample, but our results may not be fully generalizable to the Dutch population of smokers. Another limitation is that the time between Wave 8 (2014) and 9 (2015) was 16 months whereas the gap between two other waves was approximately 12 months. This may have given a slightly distorted image of the trends. Also, we used a pre-post design, which has limitations regarding internal validity: it is uncertain to what extent the introduction of PHWs has caused the observed impact since alternative explanations cannot be fully ruled out, such as secular trends or other interventions during the same period. Furthermore, the measures from this study were self-reported and respondents thus might have given socially desirable answers [43]. We aimed to prevent such social desirability responding by anonymizing the surveys. Another limitation was that the sample only included continuing smokers. PHWs may have had a larger effect on smokers who were excluded because they had quit smoking; their exclusion may have led to an underestimation of the trend after PHWs were implemented. Lastly, the measures on risk perception were somewhat limited, since we only asked about lung cancer and lung problems, and not about other health risks of smoking.

### Implications

Dutch smokers’ knowledge levels remain low as our study for instance showed that in 2017 only 67.4% of Dutch smokers knew that smoking can cause stroke and only 85.5% knew that smoking can cause lung cancer. This implies that Dutch tobacco advocacy organisations and the government should invest more in campaigns designed to improve knowledge about the health risks of smoking’. Previous research from Australia showed that media campaigns and PHWs may operate in a complementary manner to increase awareness of the health risks of smoking and quit intentions [15]. Our study showed some indication of an age and educational divide. Hence, communication policies targeted at smokers aged 55+ and low educated smokers are needed to increase awareness of the health risks of smoking. Focusing on increasing knowledge about the health risks of smoking among low educated Dutch smokers might contribute to decreasing current socioeconomic inequalities in smoking prevalence [44].

Although we cannot prove causality, the introduction of the EU’s PHWs seemed to coincide with an increase in the belief that smoking causes blindness. This finding would imply that to increase overall knowledge about the health risks of smoking, pictures may be added that cover risks from smoking that have not yet been fully communicated to the public similar to blindness. Potential risks are pregnancy complications, low bone density and hip fracture, peptic ulcer (open sores inside stomach), and diabetes mellitus type 2 [45]. In the Netherlands, emphasis should also lie on informing smokers about the health risks of secondhand smoke for non-smokers because few Dutch smokers recognized these health risks.

## Conclusions

Even after implementing EU’s PHWs, still few Dutch smokers are fully aware of the health risks of smoking. This illustrates what can happen when countries are reticent to inform smokers about these health risks. The change from THWs to the PHWs coincided with an increase in awareness of the health risks of smoking, but reconsidering the current strategy on informing smokers about these health risks with special attention to older and low educated smokers is strongly recommended.

## Data Availability

Data from the ITC Policy Evaluation Project are available to approved researchers 2 years after the date of issuance of cleaned data sets by the ITC Data Management Centre. Researchers interested in using ITC data are required to apply for approval by submitting an International Tobacco Control Data Repository (ITCDR) request application and subsequently to sign an ITCDR Data Usage Agreement. To avoid any real, potential, or perceived conflict of interest between researchers using ITC data and tobacco-related entities, no ITCDR data will be provided directly or indirectly to any researcher, institution, or consultant that is in current receipt of any grant monies or in-kind contribution from any tobacco manufacturer, distributor, or other tobacco-related entity. The criteria for data usage approval and the contents of the Data Usage Agreement are described online (http://www.itcproject.org).

## Abbreviations

CI: Confidence interval
EU: European Union
FCTC: Framework Convention on Tobacco Control
GEE: Generalized Estimating Equations
HSI: Heaviness of Smoking Index
ITC: International Tobacco Control
I-Change Model: Integrated Change Model
PHWs: Pictorial Health Warnings
SD: Standard Deviation
SPSS: Statistical Package for the Social Sciences
THWs: Textual Health warnings
TNS NIPO: Taylor Nelson Sofres Nederlands Instituut voor Publieke Opinie
WHO: World Health Organization
